# Simvastatin Attenuates Areca Nut Extract-Induced Subdermal Fibrosis in Mice by Targeting TGF-β Signaling Pathways

**DOI:** 10.3390/cimb45110542

**Published:** 2023-10-27

**Authors:** Chi-Hua Chang, Ching-Ping Lin, Yuk-Kwan Chen, Yu-Fang Hsiao, Yan-Hsiung Wang

**Affiliations:** 1 Division of Oral and Maxillofacial Surgery, Department of Dentistry, Kaohsiung Chang Gung Memorial Hospital, Kaohsiung 83301, Taiwan; xchiwha@gmail.com; 2 Division of Periodontology, Department of Dentistry, Kaohsiung Chang Gung Memorial Hospital, Kaohsiung 83301, Taiwan; jesus7286@gmail.com; 3 School of Dentistry, College of Dental Medicine, Kaohsiung Medical University, Kaohsiung 80708, Taiwan; k0285@ms22.hinet.net; 4 Division of Oral Pathology and Maxillofacial Radiology, Department of Dentistry, Kaohsiung Medical University Hospital, Kaohsiung Medical University, Kaohsiung 80708, Taiwan; 5 Oral & Maxillofacial Imaging Center, Kaohsiung Medical University, Kaohsiung 80708, Taiwan; 6 College of Medicine, Orthopaedic Research Center, Kaohsiung Medical University Hospital, Kaohsiung Medical University, Kaohsiung 80708, Taiwan; hollyhsiao@gmail.com; 7 Regenerative Medicine and Cell Therapy Research Center, Kaohsiung Medical University, Kaohsiung 80708, Taiwan; 8 Department of Medical Research, Kaohsiung Medical University Hospital, Kaohsiung Medical University, Kaohsiung 80708, Taiwan

**Keywords:** oral submucous fibrosis, areca nut extract, simvastatin, fibrosis, collagen deposition, TGF-β signaling pathway

## Abstract

Oral submucous fibrosis (OSMF) is a chronic inflammatory disease and a potentially malignant oral disorder, characterized by fibrosis of the oral mucosa. TGF-β signaling pathways have been implicated in the development of OSMF, with areca nut extract (ANE) contributing to the disease progression. Simvastatin, a statin drug, has demonstrated anti-fibrotic properties in various fibrotic conditions. However, its therapeutic potential in treating OSMF remains unclear. In this study, 8-week-old male BALB/c mice were randomly divided into three groups based on different time points. Each mouse was then treated with four different drug formulations. Post-treatment, specimens were collected for histopathological examination and staining to assess skin thickness, fibrosis, and collagen deposition. ANE treatment alone significantly increased skin thickness and collagen deposition compared to the control group after the 4-week time point. The combined administration of ANE and simvastatin, resulted in a notable reduction in skin thickness and collagen deposition. Western blot analysis revealed that simvastatin effectively suppressed the expression of fibrosis-related proteins, including CTGF, and α-SMA, in ANE-induced subdermal fibrosis. These results suggest that simvastatin has potential therapeutic effects on ANE-induced subdermal fibrosis, providing a foundation for future studies and possible clinical applications.

## 1. Introduction

Oral submucosal fibrosis (OSMF) is a disease affecting the oral mucosa, leading to restricted mouth opening and trismus [1]. OSMF is associated with poor oral hygiene and dysphagia and is considered a precursor to squamous cell carcinoma (SCC) [2]. The prevalence of OSMF is higher in the Asia-Pacific region and lower in Europe and the United States. In Manipal, India, the prevalence of OSMF among males aged 40–60 years was approximately 2% [3], while in aboriginal communities in southern Taiwan, it was approximately 17.6% [4]. The diagnosis of OSMF relies on clinical symptoms and histopathology. Clinical manifestations include palpable fibrous bands, whitening, dryness, burning, and intolerance to spicy food. Histopathological changes are divided into early, late, and moderate stages, and surgery is the main and effective treatment option [5]. Despite several drugs having been studied, there is currently no standardized drug therapy due to the lack of proven efficacy. Histopathological findings reveal an imbalance in the synthesis and degradation of extracellular matrix (ECM), primarily collagen, in the oral submucosal tissue, leading to OSMF. This imbalance leads to the overproduction of pro-fibrotic cytokines [6]. Previous studies have found that TGF-β expression is upregulated in the tissues of OSMF patients, which may play a crucial role in the development of OSMF [7,8,9]. In the fibrosis process, TGF-β plays a key role, especially in promoting the synthesis of ECM proteins which can result in excessive synthesis and collagen deposition, thus leading to the deterioration of fibrosis [8]. As a result, therapeutic strategies targeting the TGF-β signaling pathway may have potential therapeutic effects in OSMF patients [10].

Areca nut chewing is significantly and independently associated with the risk of esophageal squamous cell carcinoma [11]. The major alkaloids present in areca nut extract (ANE) have been identified as significant factors in the development of OSMF [12]. Areca alkaloids can activate epithelial cells to express TGF-β, inducing fibrosis through differentiation-related factors such as connective tissue growth factor (CTGF), endothelin, and interleukin 6 (IL-6) [13,14,15]. Additionally, ANE modulates the activity of matrix metalloproteinases (MMPs), enzymes involved in the degradation of extracellular matrix (ECM) components, leading to decreased collagen degradation and increased accumulation of ECM in fibrotic tissues [16].

Simvastatin is a member of a class of drugs known as “statins” that are primarily used for the treatment of hypercholesterolemia. Its principal mechanism of action involves the inhibition of HMG-CoA reductase, an enzyme that plays a crucial role in cholesterol biosynthesis [17]. Fibrosis is a pathological condition characterized by excessive tissue remodeling and deposition of extracellular matrix components, leading to loss of organ function [18]. OSMF is a chronic and debilitating condition associated with progressive fibrosis of the oral mucosa [19]. While simvastatin has been shown to possess anti-fibrotic properties in various fibrotic conditions, including renal [20], hepatic [21], cardiac [22], and pulmonary fibrosis [23], its effectiveness in treating OSMF is not yet clear. We established a local dermal fibrosis mouse model to simulate the pathological progression of fibrosis in OSMF. This was achieved by injecting ANE into the subcutaneous layer of the mouse back, leading to dermal fibrosis within 4 weeks. The therapeutic effect of simvastatin on OSMF is currently unclear. Thus, we established an ANE-induced OSMF model to further study the impact of simvastatin on the treatment of OSMF.

## 2. Materials and Methods

### 2.1. Chemicals and Reagents

Simvastatin was purchased from Sigma-Aldrich (St. Louis, MO, USA). ANE was obtained from Haw-Yaun Vacuum Biochemical Technology Co., Ltd. (Taoyuan, Taiwan). Antibodies against α-SMA, CTGF, total actin, and horseradish peroxidase-conjugated secondary antibodies were purchased from Cell Signaling Technology (Danvers, MA, USA).

### 2.2. Animals

To ensure the reproducibility of the OSMF status, we utilized an experimental model involving the application of ANE to the subcutaneous layer on the back of male BALB/c mice. The Animal Care and Use Committee of Kaohsiung Medical University approved all animal experiments. Nineteen 8-week-old mice with a mean body weight of 30–40 g were randomly divided into three time point groups: 1 week, 2 weeks, and 4 weeks. In the first week, each group had 6 mice, in the second week, each group had 6 mice, by the fourth week, each group had 7 mice. Additionally, four different drug formulations (groups A, B, C, and D) were applied daily to the subcutaneous layer on the back of each mouse. After the experiment, the mice were euthanized by the CO_2_ method, and specimens were collected. In addition to histopathological examination, proteins such as CTGF, Rho A, and α-SMA were analyzed by Western blot. We purchased simvastatin (10 mM) and ANE (20 mg/mL) from commercial companies and then mixed them with phosphate-buffered saline (PBS) to prepare the following four drug formulations:

(1) Group A: Simvastatin 0.05 mL + PBS 0.05 mL
(2) Group B: ANE 0.05 mL + PBS 0.05 mL
(3) Group C: Simvastatin 0.01 mL + ANE 0.05 mL + PBS 0.04 mL
(4) Group D: Simvastatin 0.05 mL + ANE 0.05 mL+ PBS 0.04 mL

Specimens were obtained from sacrificed mice and stored at −80 °C for further analyses.

### 2.3. Western Blot (WB) Analysis

Proteins were extracted from mouse tissue samples using RIPA lysis buffer containing protease inhibitors and ceramic beads, followed by homogenization using a high-speed homogenizer (model T25, IKA Labortechnik, Staufen, Germany). After centrifugation to remove tissue debris, the protein concentration in the supernatant was measured using the BCA protein assay kit (Thermo Scientific, Waltham, MA, USA). Next, protein lysates at appropriate concentrations were loaded on 10% SDS-PAGE gels and transferred to PVDF membranes. To block nonspecific binding, incubate the membrane in 1X PBS containing 5% nonfat dry milk for 1 h at 37 °C. The target protein-specific primary antibodies of anti-CTGF, anti-α-SMA and anti-β-actin were diluted appropriately and incubated overnight at 4 °C. After incubation, wash the membrane 3 times with 0.1% PBST for 10 min each, then incubated with horseradish peroxidase (HRP)-conjugated secondary antibody for 1 h at 4 °C. Bound IgG was visualized using the enhanced chemiluminescence detection kit system (PerkinElmer, Shelton, CT, USA).

### 2.4. Hematoxylin and Eosin (HE) Stain

After fixation in 10% neutral buffered formalin and paraffin embedding, 5 µm serial sections were obtained and mounted on microscope slides. The sections were deparaffinized in xylene and rehydrated through graded ethanol solutions. The HE staining protocol involved staining with Harris hematoxylin solution followed by counterstaining with eosin-fluorescein solution. The stained slides were evaluated under a light microscope to measure skin thickness, which was defined as the sum of the epidermis and dermis but not including the fat layer. Skin thickness was determined by measuring an infinite linear distance above the fat layer and averaging the values. The quantification of skin thickness was performed using the Image-Pro Plus software version 5.0. (Media Cybernetics, Inc., Bethesda, MD, USA).

### 2.5. Masson’s Trichrome Stain (MT)

Routine steps, such as fixation, grossing, processing, embedding, and sectioning, were performed before MT staining. After deparaffinization and rehydration, the sections were re-fixed in Bouin’s solution for 1 h at 56 °C to enhance the staining quality. The MT stain procedure includes staining with Weigert’s iron hematoxylin, Biebrich scarlet-acid fuchsin, and aniline blue solutions, followed by differentiation and dehydration. The tissue slides were evaluated by excluding the epidermis and hypodermis layers and hair follicles and measuring the total pixels of the dermis layer only. Collagen bundle pixels were measured by adjusting the color threshold using the O’Brien method and divided by total dermis pixels to obtain the fibrosis ratio. The fibrosis ratio was the main analytical target, and quantification was performed using Image-Pro Plus software version 5.0. (Media Cybernetics, Inc., Bethesda, MD, USA).

### 2.6. Statistical Analysis

All results, including HE and MT stains, as well as WB analyses, were presented as mean ± standard deviation. Statistical significance was determined using Friedman’s 2-way ANOVA and the Dunn post hoc test, both performed using SPSS Version 20 software. A *p*-value < 0.05 was considered statistically significant.

## 3. Results

### 3.1. Inhibitory Effect of Simvastatin on ANE-Induced Subdermal Thickening

In our study, we modified the dermal fibrosis model resulting from ANE as described by Chiang et al. [24] to explore its capacity to induce OSMF. This was achieved by administering ANE injections into the oral submucosal tissue and evaluating the ensuing outcomes. Throughout the 1–4 week treatment period, various conditions were applied to different regions on the same mouse’s back, with each region representing a distinct treatment group (Group A, B, C, or D). Skin samples were collected from each treated area. At both the 1-week and 2-week time points, no statistically significant differences were observed among the four treatment groups in terms of mean skin thickness. However, at the 4-week time point, the mean skin thicknesses were observed to be 272.57 µm for Group A, 376.20 µm for Group B, 338.23 µm for Group C, and 297.52 µm for Group D. To further investigate these differences, pairwise comparisons were conducted using Dunn’s post hoc test. This analysis revealed a significant difference between Group A and Group B (*p* = 0.003), while no significant differences were detected between Group A and the other groups (Table 1).

**Figure 1:**
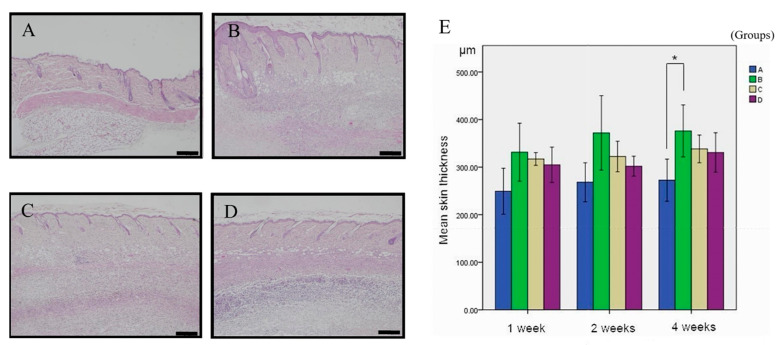
Impact of Simvastatin on Skin Thickness in ANE-Induced Subdermal Thickening. Representative images of skin thickness (HE staining) for each group at 4 weeks. (**A**) Group A (Simvastatin) exhibited baseline skin thickness. (**B**) The distance between the epidermis and dermis increased in Group B (ANE). (**C**) Skin thickness was reduced in Group C (ANE + low-dose Simvastatin). (**D**) Skin thickness was reduced in Group D (ANE + high-dose Simvastatin). (**E**): Quantitative analysis of skin thickness demonstrates a trend of reduced skin thickness with the combined use of ANE and Simvastatin. The scale bar represents 200 μm. Results were quantified using Friedman’s 2-way ANOVA and Dunn’s post-hoc test. Bars represent mean ± standard deviation; * indicates *p* < 0.05 for pairwise comparisons.

In the hematoxylin and eosin (HE)-stained sections obtained from mice at the 4-week time point, representative images of each treatment group are illustrated in Figure 1. As demonstrated in Figure 1B, treatment with ANE alone led to an increase in skin thickness in mice when compared to the control group. However, the combined administration of ANE and simvastatin resulted in a reduction of skin thickness, as observed in Panels C and D. Upon quantification of skin thickness, a statistically significant difference was observed for the group treated with ANE alone compared to the control group. In contrast, the combination of ANE and simvastatin did not yield statistically significant results (Figure 1E).

### 3.2. The Therapeutic Potential of Simvastatin on ANE-Induced Subdermal Fibrosis

At the 1-week time point, there was no significant difference in collagen deposition induced by each drug prescription. At the 2-week time point, the average fibrosis rates for Group A, Group B, Group C, and Group D were 63.47%, 84.59%, 79.92%, and 68.99%, respectively. Subsequent pairwise comparisons using Dunn’s post-hoc test revealed significant differences between Group A and Group B (*p* = 0.005) and between Group B and Group D (*p* = 0.022), while no significant differences were observed among the other groups. At the 4-week time point, the average fibrosis rates for Group A, Group B, Group C, and Group D were 63.11%, 88.21%, 75.59%, and 67.18%, respectively. Subsequent pairwise comparisons using Dunn’s post-hoc test showed significant differences between Group A and Group B (*p* = 0.005) and between Group B and Group D (*p* = 0.020), while no significant differences were observed among the other groups (Table 2).

**Figure 2:**
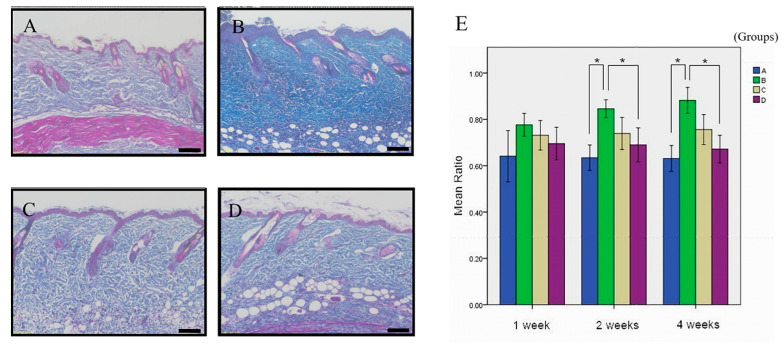
Simvastatin alleviates ANE-induced subdermal fibrosis in mice. Assessment of fibrosis ratio in mouse skin tissues under different treatment conditions at 4 weeks (MT stain). (**A**) Group A (Simvastatin) displayed normal collagen bundle distribution in the dermis. (**B**) Group B (ANE) exhibited a very thick and dense collagen bundle in the dermis due to ANE usage alone. (**C**) In Group C (ANE + low-dose Simvastatin), the collagen bundle in the dermis was thinner and looser. (**D**) In Group D (ANE + high-dose Simvastatin), the collagen bundle in the dermis was thinner and looser with the combined use of ANE and Simvastatin. The scale bar denotes 100 μm. (**E**): Quantitative analysis of the fibrosis ratio reveals a decreasing trend in fibrosis ratio with the combined use of ANE and Simvastatin. Results were quantified using Friedman’s 2-way ANOVA and Dunn’s post hoc test. Bars represent mean ± standard deviation; * indicates *p* < 0.05 for pairwise comparisons.

Previous studies have demonstrated that collagen deposition is a key characteristic of tissue fibrosis [25]. To further evaluate collagen deposition, we utilized MT staining. Collagen fibers were stained blue, allowing for quantification of collagen expression. Our findings revealed that ANE treatment alone in Group B resulted in significantly greater collagen staining compared to Group A after the 4-week time point (Figure 2A,B). Treatment with Group C and Group D formulations demonstrated decreased collagen deposition, with the Group D formulation having the most notable effect compared to Group B and reaching statistical significance (Figure 2B,D). Figure 2E displays the quantification map of collagen deposition.

### 3.3. Simvastatin Attenuates the Expression of Fibrosis-Related Factors Induced by ANE

**Figure 3:**
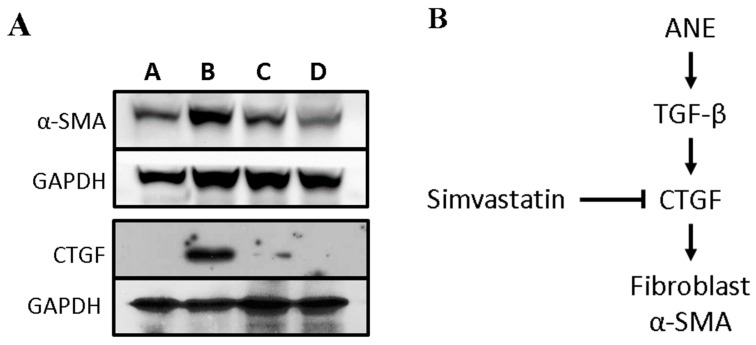
Modulation of fibrotic related proteins expression by simvastatin in ANE-treated mice. (**A**): Expression of fibrotic proteins in mice under 4 different conditions (Group A, Group B, Group C, and Group D) after 4 weeks of ANE induction. Protein expression levels of CTGF, α-SMA, and β-actin in skin tissues were detected by Western blot analysis. (**B**): A schematic diagram illustrates Simvastatin exerting anti-fibrotic effects by inhibiting the TGF-β pathways.

We subsequently investigated the potential mechanism of simvastatin’s regulatory effect on ANE-induced subdermal fibrosis. Following the 4-week time point, we collected mouse specimens and performed Western blot analysis to assess the protein levels of CTGF, and α-SMA. Compared to Group A, the administration of ANE alone in Group B resulted in a significant increase in CTGF and α-SMA protein levels. Furthermore, our results revealed that in comparison to Group B, the high-dose simvastatin-treated Group D effectively suppressed the expression of CTGF and α-SMA proteins (Figure 3A).

A schematic of the potential molecular mechanism underlying the inhibitory effects of simvastatin on fibrosis is presented in Figure 3B. This illustration provides a visual representation of the key molecular interactions and pathways involved in the process.

## 4. Discussion

Although primarily recognized as a cholesterol-lowering medication, numerous studies have unveiled its potential to inhibit fibrosis. For instance, Chung et al. reported that simvastatin could reduce capsular fibrosis following implant-based breast reconstruction, while Kuo et al. discovered its ability to attenuate cardiac fibrosis through the regulation of cardiomyocyte-derived exosome secretion [22,25]. OSMF is a precursor to oral cancer [19], and the effects of simvastatin on OSMF have yet to be clarified. Given that previous studies have established the comparison of ANE treatment with the PBS group [24,25], in this study, we aimed to investigate the impact of simvastatin on OSMF using an ANE-induced mouse model. At the 4-week time point, HE staining of the mice revealed that high-dose simvastatin significantly inhibited skin thickness. Additionally, MT staining at both the 2- and 4-week time points demonstrated a reduction in collagen density, as represented by the fibrosis ratio, in the high-dose simvastatin group. Furthermore, at the 4-week time point, Western blot analysis of the mouse samples indicated that high-dose simvastatin suppressed the expression of CTGF and α-SMA proteins. These findings collectively suggest the therapeutic potential of simvastatin in mitigating OSMF.

In the present study, we observed significant differences in skin thickness at the 4-week endpoint but not at the 1-week and 2-week endpoints (Figure 1A). This discrepancy may be attributed to several factors, including insufficient time for fibrosis induction at earlier time points, variations in cutting angles during tissue sectioning, and the influence of collagen density on skin thickness. Furthermore, the bipolar tendency of epithelium in OSMF progression could impact the results if the study were extended to longer time frames, potentially leading to thinner epidermal layers. Our findings are consistent with previous research by Chung et al., who used capsular thickness as a measure for fibrosis [26]. Figure 1B offers compelling evidence of substantial tissue structure damage under the influence of ANE for 4 weeks when compared to the control group. This pronounced alteration in structure underscores the heightened proliferation of fibroblasts and collagen accumulation—both of which are hallmark indicators of fibrosis. As depicted in Figure 1C, low-dose simvastatin appears to exert some influence on the dermal thickness and structure. However, these alterations are not statistically significant, hinting that the impact of simvastatin at low concentrations might be marginal. Consequently, it might be necessary to administer higher doses to effectively prevent or mitigate fibrosis. In stark contrast, Figure 1D unequivocally demonstrates that elevated concentrations of simvastatin significantly counteract the ANE-induced modifications in dermal tissue architecture. This lends robust support to the notion that high doses of simvastatin might be a potent therapeutic approach to address skin fibrosis. In the context of OSMF, collagen quantity and bundle diameter increase with the disease stage, while other tissue characteristics, such as blood vessels, muscle fibers, and epithelium, exhibit varying features. By focusing on skin thickness as a primary measurement, our study minimized the impact of potentially confounding factors, such as blood vessels and muscle fibers, on the results. To improve the accuracy and efficiency of our measurements, we utilized the Image-Pro Plus software version 5.0 for digital image analysis, a method first described by O’Brien et al. in 2000 [27]. As a result, we observed significant differences in fibrosis ratios, a representation of collagen density, at the 2-week and 4-week endpoints but not at the 1-week endpoint (Figure 2A). These findings align with the results obtained using HE staining and support the growing body of evidence that suggests simvastatin has the potential to reduce fibrosis in various conditions, including OSMF. Figure 2B illustrates a marked condensation and contracted state of collagen within the dermal layer, induced by ANE as revealed by MT staining. This affirms that during the fibrotic process, there’s a notable uptick in collagen accumulation within the dermis. This heightened condensation signifies more than just structural alterations in the fibrous tissue; it potentially impacts its elasticity and rigidity as well. As evident from Figure 2C,D, high-dose simvastatin effectively counters the collagen condensation and contraction triggered by ANE in the dermis. This suggests that simvastatin’s influence extends beyond regulating skin thickness; it might also modulate the structure and functionality of collagen. This bolsters its prospective efficacy in treating OSMF. While the imagery offers tangible evidence of alterations in skin tissue under different treatment regimes, delving deeper into the underlying molecular and cellular mechanisms is imperative.

TGF-β, a multifunctional cytokine, is a member of the transforming growth factor superfamily and is secreted by various cell types, including macrophages, predominantly under inflammatory conditions [9,28]. Analogous to the inflammatory response, ANE has the ability to stimulate the secretion of TGF-β by epithelial cells [15]. During processes such as cell migration, proliferation, and fibrosis, TGF-β activates downstream Smad signaling pathways and non-Smad signaling pathways (e.g., MAPK, PI3K/AKT). In a previous study by Tulek et al. [29], a single dose of intratracheal administration of bleomycin was used to induce pulmonary fibrosis in rats, followed by 14 days of intraperitoneal injection of simvastatin to evaluate its anti-inflammatory and anti-fibrotic effects in the rat pulmonary fibrosis model. In the bleomycin-induced pulmonary fibrosis group, the level of TGF-β was significantly higher compared to the control group and simvastatin group [29]. In another study by Motawi et al., rats were induced with liver fibrosis using carbon tetrachloride (CCl4) for 6 weeks, and intraperitoneal injections of MSCs and simvastatin were administered to assess their anti-fibrotic effects. In the CCl4-induced liver fibrosis group, the level of TGF-β was significantly higher compared to the control group, MSC treatment group, and simvastatin group [30]. Both studies found that TGF-β levels were significantly increased in the fibrosis groups compared to control groups and that simvastatin treatment was associated with reduced levels of TGF-β, indicating its potential anti-fibrotic effects. In our study, we did not detect the effect of simvastatin on TGF-β expression using highly sensitive experimental methods due to technical limitations. Currently, we cannot confirm the effect of simvastatin on TGF-β expression in our animal model. This result suggests that simvastatin may potentially affect TGF-β expression in mice under ANE induction. Therefore, it is necessary to further investigate whether simvastatin affects TGF-β expression in the future.

The epithelial-mesenchymal transition (EMT) is crucial in the onset of tissue fibrosis [31,32]. Both epithelial cells and stem cells possess the capability to release growth factors and cytokines, which can trigger EMT [33,34,35]. Notably, research has ascertained that ANE prompts epithelial cells to release TGFβ, subsequently fostering EMT, which can culminate in tissue fibrosis [15]. Conversely, simvastatin has been evidenced to counteract TGFβ-mediated EMT in renal proximal tubular cells [36]. Given these insights, it is reasonable to posit that simvastatin might modulate the activity of epithelial cells or stem cells, ultimately hindering the progression of EMT.

Previous studies have demonstrated that simvastatin exhibits anti-inflammatory effects by inhibiting the activity of toll-like receptors (TLRs) and mitogen-activated protein kinases (MAPKs) [37,38]. In addition, simvastatin has been found to inhibit TGF-β-induced Smad2/3 activity [39]. In human dental pulp stem cells, simvastatin has been found to promote proliferation by regulating the PI3K/AKT/miR-9/KLF5 signaling pathway [40]. CTGF is a matrix-associated protein that plays a critical role in many biological processes, including cell adhesion, migration, proliferation, angiogenesis, skeletal development, and wound healing. Most importantly, CTGF is heavily involved in fibrotic diseases [41]. Under inflammatory conditions, TGF-β has the ability to upregulate the expression of CTGF, which plays an essential role in the formation of connective tissue [8]. CTGF contributes to the formation of myofibroblasts and may further induce fibrosis and scar formation. During fibrosis and scar formation, myofibroblasts are activated and detected by α-SMA expression. Our results showed that simvastatin effectively suppressed the protein expression of CTGF and α-SMA. This finding suggests that simvastatin may directly inhibit TGF-β expression induced by ANE or participate in the regulation of TGF-β pathways. In conclusion, current research indicates that simvastatin may be a potential therapeutic agent for treating submucosal fibrosis by reducing the fibrosis ratio of the dermis.

### Limitations

This research presents some limitations that may affect the interpretation of the results. Firstly, the objective of this study was concerned with the ANE-induced mouse model to study the effect of Simvastatin on OSMF, so the control group was Simvastatin 0.05 mL + PBS 0.05 mL. A blank control group should still be included in the future to enhance the credibility of the study. In addition, due to resource constraints, only a small number of animal experiments were conducted in this study; therefore, the results of the study should be interpreted with caution, and more studies are needed to verify the underlying mechanisms.

## 5. Conclusions

In our research, we identified a notable increase in skin thickness and collagen accumulation following ANE treatment, suggesting its pivotal role in OSMF pathogenesis. However, when simvastatin was co-administered with ANE, there was a mitigating effect on these fibrotic alterations, as demonstrated by decreased skin thickness and collagen concentrations. On a molecular level, simvastatin appeared to suppress essential fibrosis-associated proteins, including CTGF and α-SMA. Our findings underscore the potential of simvastatin as a promising therapeutic candidate for combatting ANE-induced subcutaneous fibrosis, offering a prospective therapeutic approach for both OSMF research and possible clinical implementation.

## Figures and Tables

**Table 1:** The results of skin thickness measurements, defined as the distance between the epidermis and dermis, excluding the hypodermis.

Formula	1-Week (n = 6)	2-Week (n = 6)	4-Week (n = 7)
A	249.28 ± 48.34 μm	268.21 ± 41.00 μm	272.57 ± 44.41 μm ^a^
B	331.33 ± 60.99 μm	371.92 ± 78.00 μm	376.20 ± 54.66 μm ^a^
C	317.17 ± 13.53 μm	322.37 ± 32.04 μm	338.23 ± 29.06 μm ^ab^
D	304.96 ± 37.12 μm	302.03 ± 20.93 μm	297.52 ± 36.37 μm ^ab^

For each of the four variables, the same subscript letters after the mean numbers for the level designate that the levels were statistically insignificant (*p* > 0.05), different letters indicate significant differences across group comparisons (*p* < 0.05).

**Table 2:** The results of fibrosis ratio, defined as the collagen bundle ratio in the dermis, excluding the hypodermis and hair follicles.

Formula	1-Week (n = 6)	2-Week (n = 6)	4-Week (n = 7)
A	64.10% ± 11.07%	64.47% ± 5.5% ^ac^	63.11% ± 5.61% ^ac^
B	77.64% ± 4.93%	84.59% ± 3.89% ^b^	88.21% ± 5.60%
C	73.12% ± 6.35%	73.92% ± 6.92% ^abc^	75.59% ± 6.48% ^abc^
D	69.55% ± 7.03%	68.99% ± 7.32%	67.18% ± 5.96%

Identical subscript letters after mean values indicate no significant statistical difference (*p* > 0.05), while different letters signify significant variations between groups (*p* < 0.05).

## Data Availability

The data used in this study can be found within the article.
